# DNA Extraction Columns Contaminated with Murine Sequences

**DOI:** 10.1371/journal.pone.0023484

**Published:** 2011-08-18

**Authors:** Otto Erlwein, Mark J. Robinson, Simon Dustan, Jonathan Weber, Steve Kaye, Myra O. McClure

**Affiliations:** Jefferiss Research Trust Laboratories, Section of Infectious Diseases, Imperial College London, London, United Kingdom; National Institute of Health, United States of America

## Abstract

Sequences of the novel gammaretrovirus, xenotropic murine leukemia virus-related virus (XMRV) have been described in human prostate cancer tissue, although the amounts of DNA are low. Furthermore, XMRV sequences and polytropic (p) murine leukemia viruses (MLVs) have been reported in patients with chronic fatigue syndrome (CFS). In assessing the prevalence of XMRV in prostate cancer tissue samples we discovered that eluates from naïve DNA purification columns, when subjected to PCR with primers designed to detect genomic mouse DNA contamination, occasionally gave rise to amplification products. Further PCR analysis, using primers to detect XMRV, revealed sequences derived from XMRV and pMLVs from mouse and human DNA and DNA of unspecified origin. Thus, DNA purification columns can present problems when used to detect minute amounts of DNA targets by highly sensitive amplification techniques.

## Introduction

Murine endogenous retroviruses are categorised on the basis of their receptor usage and tropism and include xenotropic (x) murine leukemia viruses (MLVs) and polytropic (p) MLVs. The gammaretrovirus, xenotropic murine leukemia virus-related virus (XMRV) was first described in cases of human prostate cancer [1], [2] and chronic fatigue syndrome (CFS) [3]. Like XMRV, pMLVs sequences have been found in blood samples from CFS patients [4]. However, these findings have been challenged by others [5]–[16]. Reports describing the identification of XMRV in human tissue have also highlighted the fact that XMRV detection by PCR is unreliable and suggested that the low proviral DNA copy number accounts for this. Another explanation, however could be the detection of contaminating DNA rather than a genuine virus infection. Despite its genetic similarity to xMLVs [1], XMRV has no reservoir in mice [17], but several copies of the virus are present in the human prostate cancer cell line, 22Rv1, which releases infectious virus particles [18]. It has been proposed that XMRV was generated in the 1990s from a unique recombination event between two murine endogenous proviruses when the 22Rv1 cell line was being established, a process that involved repeated passages through nude mice [17]. The authors also point out that since it is highly unlikely that a similar recombination event occurred elsewhere by random chance, then all XMRV isolates of similar sequence owe their provenance to this event and, hence, there is no natural human reservoir of XMRV.

Detection of XMRV and other MLV-related viruses, generally rely on PCR amplification of integrated proviral DNA sequences. It is, therefore, a source of concern that several XMRV publications have described the ease with which samples can be contaminated with murine DNA [19]–[22]. One highly sensitive way to distinguish between mouse and murine retroviral DNA is to look for intracisternal A particle (IAP) sequences, retrotransposons present at about 1,000 copies per mouse genome [23].

## Analysis

### Detection of Murine DNA in FFPE Columns

Using Qiagen's QIAamp DNA FFPE Tissue Kit (Qiagen, Crawley, UK) as directed in the manufacturer's instructions, we recently observed that DNA extracted from formalin-fixed paraffin-embedded (FFPE) prostate cancer tissue from the UK, Thailand and Korea (supplied from non commercial sources as detailed in [19]), occasionally contained mouse DNA. This murine contamination was detected by PCR using primers IAP forward and IAP reverse (Table 1), specifically designed to amplify IAP sequences. We used TaqGold polymerase (Applied Biosystems, Warrington, UK) for the PCR under described conditions [19].

**Table 1:** DNA primer sequences.

Name	Sequence	Position *
XMRV Forward outer	5′ CATTCTGTATCAGTTAACCTAC 3′	411–432
XMRV Reverse outer	5′ ATGATCTCGAGAACACTTAAAG 3′	588–609
XMRV Forward inner	5′ GACTTTTTGGAGTGGCTTTGT 3′	411–461
XMRV Reverse inner	5′ ACAGAAGAACAACAAAACAAATC 3′	544–566
XMRV-R	5′ GGGCCAGTCATCCGATAGACT 3′	8109–8129
XTP1	5′ CACCCACTCTTTCCTCCATGT 3′	2437–2457
MLV reverse outer	5′ CATCAAACAGGGTGGGACTG 3′	3160–3179
1154R	5′ GCCGCCTCTTCTTCATTGTTCTC 3′	1127–1149
5922F	5′ GCTAATGCTACCTCCCTCCTGG 3 ′	5917–5938
6273R	5′ GGAGCCCACTGAGGAATCAAAACAGG 3′	6242–6267
IAP forward	5′ ATAATCTGCGCATGAGCCAAGG 3′	
IAP reverse	5′ AGGAAGAACACCACAGACCAGA 3′	
IAP PROBE	5′ FAM-ATGGGCTGCAGCCAATCAGGGAGTGAT-TAMRA 3′	

*GenBank accession no. EF185282.1.

Sequences for IAP were obtained from elution buffer processed through several empty (control) columns used directly as supplied by Qiagen. Multiple PCR water controls (not processed through the columns) were consistently negative, indicating that the PCR buffers and polymerase enzyme used in the PCR were clean and that mouse contamination had not been introduced during the reaction (data not shown).

**Figure 1:**
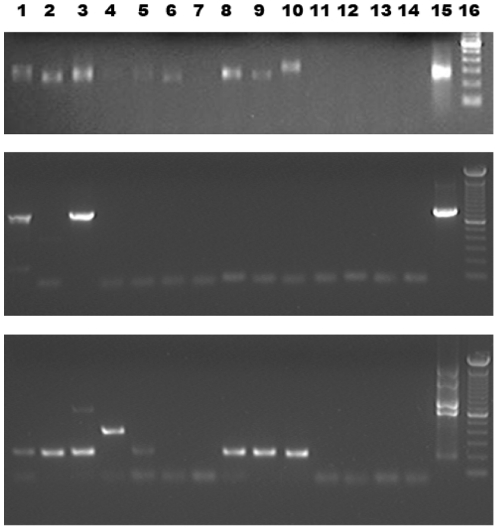
Amplification of contaminating DNA from empty columns of the QiaAmp FFPE Tissue Kit. Lanes 1–10, naïve DNA extraction columns; lanes 11–14, PCR water controls; lane 15, positive control; upper panel, McCoy cellular DNA; middle and lower panel, XMRV VP62 infectious clone; lane 16, 100 bp DNA ladder (Invitrogen, Paisley, UK). Upper panel, detection of contaminating sequences using IAP-specific primers IAP for and IAP rev. All columns apart from column no 7 produce amplicons. Size differences reflect the fact that IAP sequences form a class of slightly different retrotransposons. Middle panel, PCR products using primers XTP1 and MLV reverse outer under relaxed annealing conditions. Lower panel, multiplex PCR using the four primers XMRV-R, XMRV Forward outer, XMRV Reverse outer and 1154R under less stringent annealing conditions.

It was not possible to determine if the kit buffers were contaminated with mouse DNA, as they inhibited the IAP PCR when they were added to the positive control, namely DNA from McCoy murine fibroblast cell line (ECACC #90010305), (data not shown). To investigate the prevalence of murine DNA signals in FFPE columns we looked at a total of 68 “mock-extracted” column eluates following manufacturer's instructions, but without adding any tissue sample. We detected signals for IAP in 11 of these 68 columns assayed (16.1%) (Fig. 1, upper panel and data not shown). The PCR product shown in Fig. 1, lane 10 was cloned into plasmid pPCR4TOPO (Invitrogen, Paisley, UK), sequenced and identified as IAP.

### Detection of XMRV and pMLV Sequences in FFPE Columns

As recently described [19] DNA extracted from IAP positive prostate cancer tissue occasionally also gave rise to pMLV sequences and XMRV-specific PCR products, the latter defined by the 24 bp deletion in the leader/*gag* region of XMRV [19]. When nested PCR was carried out on the eluate of the 11 IAP-positive mock-extracted columns, a fragment with a sequence identical to XMRV was amplified from one of them. The nested PCR conditions to detect XMRV sequences have been documented [7]. First-round reaction conditions were the same as described for the amplification of IAP sequences, but with primers XMRV Forward outer and XMRV Reverse outer (Table 1). In order to clone and sequence the PCR product, the second round again made use of the XMRV Forward outer and XMRV Reverse outer primers, as these bind outside the XMRV-specific 24 bp deletion. The XMRV infectious clone, VP62 plasmid DNA, constituted the positive control. Using less stringent annealing conditions (50°C instead of 55°C), a PCR with primers XTP1 and MLV reverse outer, which targets the XMRV *gag/pro/pol* open reading frame (ORF), amplified further products (Fig. 1, middle panel). Under the same conditions, a multiplex PCR with the four primers XMRV-R, XMRV Forward outer, XMRV Reverse outer and 1154R (Table 1) that bind to the long terminal repeat, the leader/*gag* sequence and the *gag* ORF, also produced various amplicons (Fig. 1, lower panel). Using published primers to the XMRV *env* region [3], three out of ten columns tested produced an amplification product (not shown). At least four “no template” water controls were included in each experiment.

**Figure 2:**
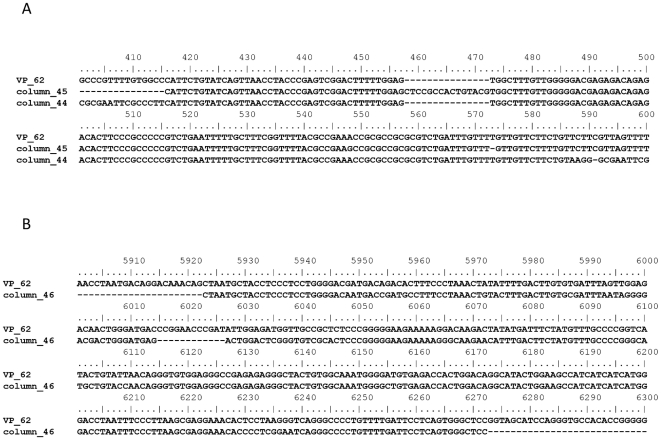
Alignment of the infectious molecular clone of XMRV, VP 62, and sequences obtained from mock eluted columns. (A) Alignment of the leader/*gag* region, displaying the XMRV specific deletion. (B) Alignment of the *env* region.

Several of these amplicons were cloned and sequenced. A GenBank database search indicated contaminating sequences of human and murine origin. These included two leader/*gag* regions displaying the 24 nt deletion described for XMRV (one sequence being identical, one having two mismatches and a 1 bp insertion, respectively), five leader/*gag* regions of pMLVs, all displaying a 9 bp deletion, one *gag/pol* ORF of pMLV or XMRV (8 mismatches and 10 mismatches out of 599 bp, respectively), and one sequence of the *env* gene of a murine enogenous polytropic retrovirus. In addition one DNA sequence had homology to mouse chromosome 11, two other DNA sequences showed homology to human chromosome 6 and 10. Two further sequences were of unspecified origin. Although these were not mapped to any particular gene, they are nevertheless an indication of contaminating DNA from a variety of sources. Alignments of leader/*gag* and *env* sequences obtained from the columns and VP62, the reference XMRV infectious clone (GenBank accession no. EF185282.1), are shown in Fig. 2.

### Detection of Murine DNA in Other Columns

Columns of the QIAamp DNA Mini Kit were also mock-extracted as described in the manufacturer's instructions, but no IAP sequences were identified in the eluate. In a further experiment, carried out by a different operator in a separate laboratory, a column from the QiaAmp DNA Investigator Kit was dismantled. After soaking the DNA binding membrane in elution buffer overnight, samples of this elution buffer were amplified in a real time PCR (RT-PCR) specific for IAP. The RT-PCR was carried out using the Quantitect probe kit (Qiagen, UK) as per manufacturer's instructions, using the primers and probes described in Table 1. Reactions were in volumes of 10µl to which 2.5µl eluate was added and were amplified in a Biorad CFX96 thermal cycler. Qiagen 2x Quantitect probe mastermix was used (Qiagen, UK) with 2.5 pmol of each primer IAP for and IAP rev. In addition, 2.5 pmol IAP PROBE was added to each reaction. Cycling conditions were one cycle of 95°C 15 seconds followed by 60 cycles of 94°C 15 seconds, 60°C 15 seconds. As a positive control, DNA from McCoy cells was used. At least 6 “no template” controls were set up in each RT-PCR. The control sample of unexposed elution buffer remained negative, but an IAP-specific signal from elution buffer exposed to the column pieces was observed. Upon cloning, the sequence of the amplicon was confirmed to be IAP (data not shown). It is worth noting that QIAamp Ultraclean Production (UCP) Pathogen columns which are certified to be free of contaminating microbial DNA yielded no amplification product for IAP or MLV-related sequences in 50 “mock-extracted” columns.

## Discussion

There are many commercially available kits that rely on DNA binding columns to extract and purify DNA from tissues or cultured cells. Our observations by two different laboratory investigators (OE and MJR) using three different kits and working in separate laboratories, demonstrate that they can be contaminated with DNA of diverse provenance. This includes DNA from mice. It cannot be ruled out that some of the buffers used during the DNA extraction process were contaminated and, in turn, resulted in contamination of some of the columns. We tested the elution buffers from several kits and found no evidence for contamination. A confounding issue was the fact that tissue lysis buffers and washing buffers in these kits were found to contain substances that inhibited the PCR and, therefore, the buffers could not be reliably tested. Therefore, it is possible that these buffers contain traces of DNA which bind to the columns and are eluted in later steps, contaminating the sample. However, it is telling that dismantled column parts soaked in elution buffer resulted in an IAP signal while the elution buffer control did not, suggesting that the columns themselves can be contaminated.

Recently, several publications documented that widely used PCR enzymes and buffers can be contaminated with murine DNA [13], [14], [24]. Taken together with the data presented here, these results may explain some of the spurious “detections” of XMRV or related pMLV sequences [2], even in laboratories that use neither mice nor XMRV-infected cell lines and avoid enzymes known to contain traces of murine DNA. For those involved in detecting minute amounts of retroviral sequences in human tissue, these data may serve as a useful reminder to check reagents to confirm that murine sequences are absent before analysing tissue samples.
